# Prerequisites of Third-Person Pronoun Use in Monolingual and Bilingual Children With Autism and Typical Language Development

**DOI:** 10.3389/fpsyg.2019.02289

**Published:** 2019-10-15

**Authors:** Natalia Meir, Rama Novogrodsky

**Affiliations:** ^1^Department of English Literature and Linguistics, Bar-Ilan University, Ramat Gan, Israel; ^2^Department of Communication Sciences and Disorders, University of Haifa, Haifa, Israel

**Keywords:** high-functioning autism, bilingualism, pronoun use, Theory of Mind, morpho-syntax, working memory, inhibition

## Abstract

The current study investigated the production of third-person subject and object pronouns in monolingual and bilingual children with High Functioning Autism (HFA) and typical language development (TLD). Furthermore, it evaluated the underlying linguistic and non-linguistic prerequisites of pronoun use, by assessing the role of morpho-syntactic skills, Theory of Mind (ToM) abilities, working memory and inhibition on pronoun use. A total of 85 children aged 4 to 9 years participated in four groups: 27 children with HFA [14 monolingual (monoHFA) and 13 bilingual (biHFA)], and 58 children with TLD [28 monolingual (monoTLD) and 30 bilingual (biTLD)]. All children spoke Hebrew and the bilingual children spoke Russian as their Heritage Language. Third-person subject and object pronouns were elicited in Hebrew. The results yielded no effect of bilingualism, and a robust effect of HFA on the use of pronouns. Bilingual Russian-Hebrew speaking children paired up with their monolingual Hebrew-speaking peers in pronominal use in Hebrew. Monolingual and bilingual children with TLD showed nearly ceiling performance on pronoun use. The facilitative effect of pronominal acquisition in Hebrew among bilingual children was attributed to similarities in the pronominal systems of the two languages of bilingual children. Age was found to be a predictive factor of pronoun use in children with TLD. Conversely, children with HFA had a lower rate of pronoun production compared to the TLD groups. Both third-person subject and object pronouns were largely predicted by morpho-syntactic abilities of children with HFA. In addition, subject pronoun use was predicted by ToM skills and working memory confirming that pronoun use is a complex phenomenon, which requires integration of multiple linguistic and non-linguistic components. To conclude, our findings suggest that morpho-syntactic development is a prerequisite for third-person subject and object pronoun use in children with HFA, and ToM and working memory are involved in third-person subject pronoun use. In addition, we show that pronoun use is not compromised by dual language exposure in children with TLD and with HFA.

## Introduction

The current study explored the use of Hebrew third-person subject and object pronouns in monolingual Hebrew-speaking and bilingual Russian-Hebrew speaking children, in a subgroup of children with Autism Spectrum Disorder (ASD), i.e., children with High Functioning Autism (hereafter HFA) who have non-verbal IQ scores within the normal range, and in children with typical language development (TLD). We also assessed the underlying linguistic and non-linguistic prerequisites of pronoun use, by looking into the role of morpho-syntactic skills, Theory of Mind (ToM) abilities, working memory and inhibition on pronoun use. Bilingual children with and without HFA in the current study were acquiring Hebrew as their Societal Language and Russian as their Heritage Language.

A pronominal element (e.g., *I, you, he, she, it*) is used in place of a noun phrase that has already been mentioned or that is already known, often to avoid repeating the noun. Languages differ with respect to how they express pronominal elements^1^ : full pronouns (strong and weak) and clitic pronouns (Cardinaletti and Starke, 1996). Furthermore, languages differ whether and how they license null pronominal elements^2^. Despite being typologically different languages, Hebrew and Russian show some similarities in their pronominal systems. Pronominal elements of Hebrew and Russian are presented in greater detail in subsection 1.3.

Pronoun use is a complex linguistic phenomenon as it represents the interface between linguistic (e.g., morpho-syntactic knowledge and discourse-pragmatic knowledge) and non-linguistic capacities (e.g., ToM skills and working memory) (for an overview see Sorace et al., 2009). First, in many languages including Hebrew, which was investigated in the current study, third-person subject and object pronoun use requires the integration of morpho-syntactic components such as distinctions of gender (*he, she*), number (*he, they*), person (*he, you*) and case (*he, him*). Second, discourse-pragmatic knowledge, which regulates the distribution of overt versus null pronominal elements, is essential in languages in which null pronominal elements are allowed (e.g., Grimshaw and Samek-Lodovici, 1998; Ariel, 2001, 2004). Furthermore, pronominal use requires understanding of other people’s mental states in order to evaluate the interlocutor’s knowledge and whether or not a referent is familiar to the listener or not (e.g., Gundel et al., 2006; Arnold et al., 2009). Mentalizing others’ states can require potentially inhibiting one’s own perspective. Finally, limited working memory capacity has been shown to be associated with poor pronoun use (Koster et al., 2011) and comprehension (Marinis and Chondrogianni, 2011).

The motivation for investigating subject and object pronoun use in monolingual and bilingual children with and without HFA stemmed from two bodies of literature. First, studies on the production of pronominal elements in populations with ASD/HFA bring inconclusive evidence (Marinis and Chondrogianni, 2011; Chondrogianni, 2015). Secondly, results on pronoun use in bilingual children as compared to monolingual children are also mixed (Sorace et al., 2009). Thus, we aimed to contribute to the literature on how bilingualism and ASD, separately and in combination, affect third-person subject and object pronoun use. Beyond the language status (monolingual vs. bilingual) and developmental disorder (HFA vs. TLD), the current study explored three underlying mechanisms that potentially affect pronoun use: morpho-syntactic abilities, ToM skills, and executive functions (working memory and inhibition).

### Subject and Object Third-Person Pronoun Use in Children With ASD/HFA

In this subsection, we will discuss the available evidence on the production of third-person pronominal elements in subject and object positions in children with ASD and will present the underlying mechanisms, linguistic and non-linguistic, associated with pronominal production in this clinical population.

Autism Spectrum Disorder is a complex neurobiological disorder of early development. ASD is diagnosed on the basis of two symptom clusters: (1) pervasive deficiencies in social communication and social interaction, and (2) restrictive and repetitive patterns of behavior, interests or activities (the Diagnostic and Statistical Manual of Mental Disorders-5; American Psychiatric Association, 2013). The term HFA is applied to individuals with ASD who have non-verbal IQ scores within the normal range (Bishop, 2003). Language is not part of the ASD diagnosis, and there is no agreement on the relations between language and cognitive abilities of children with ASD (Tager-Flusberg, 2016). Research on pronominal elements in children with ASD is of high importance as it is at the interface of linguistic (morpho-syntax and discourse-pragmatics) and non-linguistic knowledge. Deficits in pragmatics and discourse are characteristic features of ASD linguistic phenotype (for an overview see Eigsti et al., 2011). With respect to morpho-syntax, previous research has shown that some children with ASD have a comorbid Language Disorder, while some develop intact morpho-syntactic skills (Durrleman and Delage, 2016; Meir and Novogrodsky, 2019; Kjelgaard and Tager-Flusberg, 2001).

Research on the production of third-person subject and object pronominal elements in monolingual children with ASD provide mixed evidence (Marinis and Chondrogianni, 2011; Chondrogianni, 2015). There are studies showing that children with ASD have problems with production of third-person pronominal elements (Tager-Flusberg, 1995; Novogrodsky, 2013; Durrleman and Delage, 2016; Novogrodsky and Edelson, 2016; Tuller et al., 2017; Prévost et al., 2018; Sukenik and Friedmann, 2018). Production of clitic pronouns is reported to be impaired in a subgroup of children with ASD, who have a comorbid Language Disorder, similarly to children with Developmental Language Disorder (Durrleman and Delage, 2016; Tuller et al., 2017; Prévost et al., 2018). Conversely, there are studies showing that children with ASD produce third-person pronominal elements at the same rate and accuracy as children with TLD (Tager-Flusberg, 1995; Arnold et al., 2009; Terzi et al., 2019).

In addition to linguistic aspects that hinder the use of pronominal elements in children with ASD, several non-linguistic components (e.g., ToM skills, working memory capacity, and inhibition) have been associated with poor use of pronounal elements. The use of pronominal elements has been linked to ToM skills, the ability to mentalize about other people’s knowledge. Impaired ToM is a core feature of ASD (Baron-Cohen et al., 1985; Baron-Cohen, 1988, 2000; Yirmiya et al., 1998). Furthermore, working memory capacity and ability to inhibit your own perspective have been suggested to be implicated in pronoun use; these cognitive capacities have been reported to be impaired in children with ASD (Hill, 2004; Martinussen et al., 2005). For example, Durrleman and Delage (2016) showed that in addition to intact versus impaired morpho-syntactic skills, the production of first-person object clitic pronouns in monolingual French-speaking children was related to ToM skills, while the production of third-person clitics was related to working memory. Slightly different findings were reported by Kuijper et al. (2015) who found that the use of third-person ambiguous referential expressions was related to ToM, and to a lesser degree to inhibition and working memory capacity.

The current study aimed to explore the role of linguistic and non-linguistic factors on the use of third-person subject and object pronoun not only in monolingual children with HFA, but also in bilingual children. Studies on bilingual children with ASD are rare. The lack of knowledge of this specific population creates barriers for professionals encountering bilingual children with ASD (Welterlin and LaRue, 2007; Yu, 2013). In the next subsection, we discuss available evidence on the effect of bilingualism on the use of third-person pronominal elements in children with TLD.

### Subject and Object Third-Person Pronoun Use in Bilingual Children With TLD

Evidence on pronominal use in bilingual children is mixed. On the one hand, there are studies demonstrating that bilinguals do not differ from monolinguals in the production of pronominal elements. Conversely, there are studies showing that exposure to two languages might affect children’s and adults’ production of pronouns (for an overview see Sorace, 2016). As suggested above, pronoun use requires the integration of linguistic and non-linguistic components. A number of studies have demonstrated that the interface of morpho-syntax and discourse is vulnerable in bilingual speakers (Hulk and Müller, 2000; Paradis and Navarro, 2003; Sorace, 2005; Serratrice, 2007; Sorace and Serratrice, 2009).

Some studies focused on morpho-syntactic features of pronoun acquisition (for an overview see Prévost, 2015) showing delayed acquisition of pronominal elements, especially when the lexical realization (e.g., clitic pronouns vs. strong/weak pronouns) and the placement (pre-verbal vs. post-verbal) of pronominal elements differ in the two languages of a bilingual child. Bilingual children seem to omit pronominal elements in object positions and have problems with gender/number/person features and placement of clitic pronouns. However, there is also evidence that bilinguals perform on par or even outperform monolinguals on object pronominal elements. For example, Bilingual French-English speakers showed a higher rate of strong pronouns than monolinguals did in object positions in French; this advantage was attributed to their exposure to the English pronominal system, which uses full pronouns (Paradis et al., 2006).

Previous studies showed that bilingual children learning a subject [+pro-drop] language in tandem with a subject [-pro-drop] language overuse overt subject pronouns in their [-pro-drop] language (Paradis and Navarro, 2003; Serratrice et al., 2004; Hacohen and Schaeffer, 2007). The overt use of subject pronouns by bilinguals who speak languages that do not allow subject pro-drop (e.g., English) and languages that allow subject pro-drop (e.g., Italian, Greek) could be attributed to the influence of a Heritage Language that does not allow subject pro-drop.

Alternatively, bilinguals are suggested to overextend the option that monolinguals employ and their use of pronouns is “over-explicit” (Sorace, 2016). “Over-explicitness,” i.e., over-production of overt pronouns in contexts in which monolingual speakers use null elements, is linked to enhanced ToM skills in bilinguals, which result in a higher threshold for deciding which reduced form is unambiguous (Sorace, 2016; Schroeder, 2018). For example, a recent study showed that bilingual children with Developmental Language Disorder used fewer ambiguous third-person pronouns than the monolingual peers did (Tsimpli et al., 2016). The authors reported a link between the use of pronouns and ToM skills, suggesting that ToM capacity explains a bilingual advantage in third-person pronouns.

To conclude, beyond linguistic factors, i.e., cross-linguistic similarities/differences between the two languages of bilingual children, non-linguistic components (e.g., ToM) might affect the use of pronouns in bilingual children. The next subsection will overview the pronominal systems of Hebrew (the Societal Language of all the children tested in the study) and Russian (the Heritage Language of the bilingual children in the current study) in order to determine whether the differences/similarities in the use of pronouns in Hebrew among bilingual Russian-Hebrew speaking children might be linked to cross-linguistic influence.

### Third-Person Subject and Object Pronouns: Comparison of Hebrew and Russian

Pronominal systems vary across languages: some languages use clitic pronouns, some languages use strong and weak pronouns. Furthermore, languages vary with respect to licensing null pronominal elements. Despite being typologically different languages, Russian and Hebrew show some similarities in their pronominal elements. Both languages use full pronouns, which are inflected for number (Russian: *on* “he” vs. *oni* “they”; Hebrew: *hu* “hi” vs. *hem* “they”), gender (Russian: *on* “he” vs. *ona* “she”; Hebrew: *hu* “hi” vs. *hi* “she”), and case (Russian: *on* “he” vs. *ego* “him”; Hebrew: *hu* “hi” vs. *oto* “him”). Both Russian and Hebrew lack pronominal clitics, and in both languages weak and strong personal pronouns are homophonous (for Hebrew see Laenzlinger and Shlonsky, 1997; for Russian see Testelets, 2003).

Furthermore, Russian and Hebrew have subject-verb agreement and rich verbal inflectional paradigms. On the other hand, neither language has object-verb agreement. We will discuss below that the two languages show a number of similarities with respect to licensing of null pronominal subject and object.

#### Third-Person Subject Pronouns in Hebrew and in Russian

Unlike traditional pro-drop languages (e.g., Italian, Spanish), which allow omissions of subject pronouns, and traditional non-pro-drop languages (e.g., English), which require overt subject pronouns, Hebrew shows a mixed pattern. Hebrew is labeled as a partial pro-drop language presenting a complex pattern of licensing subject null pronominal elements (Melnik, 2007; Holmberg et al., 2009; Shlonsky, 2009). While first- and second-person pronominal null elements in past and future tense are conditioned by subject-verb agreement (in these cases overt pronouns are used for emphasis and contrast), pro-drop is not possible with third-person subject pronominals in the past and in the future, and it is not possible in all cases of present tense.

Although third-person subject pro-drop is not licensed by the person feature, it is possible in Hebrew in adjunct subordinate clauses (Melnik, 2007). Furthermore, third-person null subjects in Hebrew are possible under the discourse-drop condition, which is illustrated in (1): in such cases, an antecedent is contextually (linguistically or situationally) available.


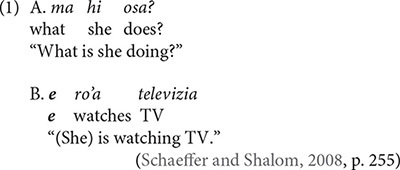


Similarly, to Hebrew, Russian presents a mixed pattern with respect to null subjects. There is no agreement on the classification of Russian with respect to subject pro-drop. Russian is labeled as a non-pro-drop language (Franks, 1995), or a partial pro-drop language (Barbosa, 2011). Third-person subject pro-drop is not licensed by morpho-syntax, yet, similarly to Hebrew, null subjects are possible in subordinate clauses if a pronominal element is co-indexed with a lexical noun phrase in the matrix clause (e.g., Ivanova-Sullivan, 2014). Furthermore, similarly to Hebrew, Russian allows null pronominal subjects under the discourse-drop condition (see Gordishevsky and Avrutin, 2003).

#### Third-Person Object Pronouns in Hebrew and in Russian

Turning to object pronouns, Hebrew does not have object-verb agreement, as it has been previously discussed, thus object drop is not licensed morpho-syntactically. On the other hand, there is evidence that under discourse-drop conditions, omissions of object pronouns are possible [see (2)] (Doron, 1999; Landau, 2018). Similar to Hebrew, Russian allows null pronominal object elements under the discourse-drop condition.


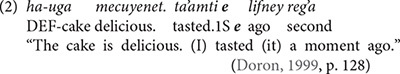


Thus, despite being typologically different languages, Russian and Hebrew show some similarities with respect to the use and drop of third-person subject and object pronouns. Both languages are partial subject pro-drop languages, showing a mixed pattern of licensing of null subject elements based on morpho-syntactic features. Both languages license third-person subject and object null elements based on discourse conditions. Thus, the similarities between the two languages might facilitate the use of pronouns in bilingual Russian-Hebrew children.

### Acquisition of Pronouns in Hebrew-Speaking Children

Having presented the pronominal systems of Hebrew and Russian, we will provide an overview of the available studies on the production of third-person subject and object pronouns in Hebrew-speaking monolingual and bilingual children with TLD.

Based on spontaneous child data in Hebrew, pronouns are reported to be productively used in subject and object positions at ages 2;0 to 2;6 years (Berman, 1985; Rom and Dgani, 1985; Armon-Lotem, 2008; Berman and Lustigman, 2012). In the same vein, the results from elicited production experiments showed very high rates of third-person object pronoun production and low rates of omissions (Ruigendijk et al., 2010; Varlokosta et al., 2016). The adult data show that third-person object pronouns are produced in 98% of contexts, full noun phrases are found only in 0.8% of cases, null elements are not attested (Varlokosta et al., 2016).

Turning to bilingual children who speak Hebrew as their Societal Language, Hacohen and Schaeffer (2007) reported higher use of overt subject pronouns in a English-Hebrew bilingual as compared to monolinguals, suggesting that over-use of overt subject pronouns in Hebrew (a partial pro-drop language) is attributed to the properties of English, which does not license null subject pronouns. Looking into Russian-Hebrew bilinguals, a recent paper by Fichman and Altman (2019) explored the production of referential expression (i.e., full noun phrases, pronouns and null elements) in narratives of monolingual and bilingual children with and without Developmental Language Disorder in Hebrew and in Russian. Although, bilinguals and monolinguals were not directly compared, the data show that the use of full noun phrases, pronouns and null pronominal element was similar in Hebrew for Hebrew monolinguals and Russian-Hebrew bilinguals.

To sum up, previous studies show that the production of subject and object third-person pronouns in monolingual Hebrew-speaking children is target-like by the age of 5–6. In bilingual acquisition, differences might be attested in children whose Heritage Language shows different principles of null-element licensing [as in the case of English-Hebrew bilinguals in the study by Hacohen and Schaeffer (2007)]. Yet, in the case of bilinguals whose languages show a similar pattern of null-versus-overt pronoun use, differences might not be observed [as in the case of Russian-Hebrew bilinguals in the study by Fichman and Altman (2019)].

### The Current Study

The current study was devised to explore separate and combined effects of HFA and bilingualism on third-person subject and object pronoun use. Based on previous literature, third-person subject and object pronoun use might be compromised in children with HFA as it has been previously reported in some studies (not all). Yet, in the case of bilingual children with TLD, we expected to find no effect of bilingualism. Specifically, no differences between monolinguals and bilingual children with TLD were expected based on the similarities in the pronoun realization and licensing of null pronominal element in the two languages of the bilinguals in the current study.

Furthermore, the study aimed to contribute to the literature by evaluating the underlying mechanisms implicated in the use of third-person subject and object pronouns (e.g., morpho-syntactic skills), mentalizing skills (e.g., ToM skills), and executive functioning skills (e.g., inhibition and working memory).

## Materials and Methods

### Participants

A total of 85 children were recruited for this study: 27 children with HFA [14 monolingual (monoHFA) and 13 bilingual (biHFA)], and 58 children with TLD [28 monolingual (monoTLD) and 30 bilingual (biTLD)]. All children spoke Hebrew.

#### Bilingual Children

Bilingual children with HFA and TLD were matched for their Heritage Language (Russian). All bilingual children were born to Russian-speaking parents. At the time of testing, they were attending mainstream or special communication pre-schools/schools in which Hebrew was the language of instruction. In Israel, compulsory education starts at the age of three, and children spend 5–6 days a week (varies across the country, based on local rules) from 8:00 until 13:00 in these educational settings. In special communication pre-schools/schools, the school day is longer, till 16:00 or 17:00. Background information was collected via the “BIPAQ” parental questionnaire for bilingual children and “MONOPAQ” parental questionnaire for monolingual children (Abutbul-Oz et al., 2012). In addition, we administered Raven’s colored progressive matrices (Raven, 1998) as a measure of non-verbal IQ.

#### Children With TLD

Children with TLD (monolingual and bilingual) had no prior parental concerns about their language milestones and did not have any diagnosed developmental disorders such as Development Language Disorder, ASD, hearing impairment and/or attention hyperactivity deficit disorder, as determined by parental questionnaires. All children with TLD were attending mainstream kindergartens and schools.

#### Children With HFA

Children with HFA (monoHFA and biHFA) were diagnosed prior to the study and were recruited from special education kindergartens and classes for children with communication disorders. As part of our assessment battery, we administered the Autism Diagnostic Observation Schedule (ADOS, Lord et al., 2003) to all children with autism to reconfirm their diagnosis for research purposes.

#### Demographic Data on the Participants

Table 1 presents background information of the participants in each group. A one-way ANOVA showed that the groups were matched for chronological age [*F*(3,81) = 0.05, *p* = 0.98] and non-verbal IQ [*F*(3,81) = 1.16, *p* = 0.33]. A one-way ANOVA indicated group differences for SES as measured by mother’s years of education [*F*(3,81) = 3.30, *p* = 0.02], yet none of the *post hoc* pair-wise comparisons reached significance (*p* > 0.05). The monoHFA and biHFA groups showed no significant differences in the severity of autism as measured by ADOS scores [*t*(25) = 1.44, *p* = 0.16]. The two bilingual groups did not differ in age of onset of the Societal Language [*t*(41) = 0.26, *p* = 0.80], length of exposure (calculated as the child’s chronological age minus age of onset of Hebrew) [*t*(41) = 0.01, *p* = 0.99], and current exposure to Hebrew [*t*(41) = 0.29, *p* = 0.77].

**TABLE 1:** Background information of the participants in each group.

**Variable**		**monoHFA (*n* = 14)**	**biHFA (*n* = 13)**	**monoTLD (*n* = 28)**	**biTLD (*n* = 30)**
Gender (girls/boys)		0/14	2/11	18/10	16/14
Age (months)	*M* (*SD*)	80 (19)	82 (17)	81 (13)	80 (13)
	Range	54–110	60–108	63–100	60–103
Mothers’ education (years)	*M* (*SD*)	15 (2)	15 (4)	16 (2)	18 (3)
	Range	12–18	12–25	12–21	10–24
Raven (raw score)	*M* (*SD*)	20 (6)	22 (7)	23 (7)	24 (6)
	Range	13–32	13–36	10–34	14–36
ADOS (raw score)	*M* (*SD*)	12 (4)	10 (2)	n/a	n/a
	Range	8–21	7–14		
Age at onset of the Societal Language (months)	*M* (*SD*)	n/a^1^	18 (27)	n/a^1^	16 (18)
	Range		0–80		0–60
Length of exposure to the Societal Language (months)	*M* (*SD*)	n/a^1^	64 (26)	n/a^1^	64 (30)
	Range		19–108		11–96
Current exposure to the Societal Language (%)	*M* (*SD*)	n/a	54 (14)	n/a	53 (14)
	Range		25–75		25–75

*^1^For monolingual Hebrew-speaking children, age of onset is 0 (i.e., from birth), since they were not exposed to any other language and length of exposure to the Societal Language (Hebrew) is equal to their chronological age.*

#### Background Measures (Morpho-Syntax, ToM, Working Memory, and Inhibition)

We compared the four groups of children on the following background measures: morpho-syntactic abilities, ToM Skills and Executive Functioning (working memory and inhibition) (Table 2). It should be noted that monolingual and bilingual children with HFA showed considerable heterogeneity in performance on all four measures (see range scores for monolingual and bilingual children with HFA in Table 2).

**TABLE 2:** Performance per group on linguistic and cognitive measures.

		**Mono HFA (*n* = 14)**	**Bi HFA (*n* = 13)**	**Mono TLD (*n* = 28)**	**Bi TLD (*n* = 30)**	***F*-value**	***p-*value**	**Tamhane *post hoc***
Syntactic abilities	*M* (*SD*)	0.63 (0.24)	0.58 (0.26)	0.96 (0.06)	0.85 (0.15)	21.01	<0.001	(monoHFA = biHFA)
(maximum score 1)	Range	0.03–0.93	0.17–0.93	0.83–1.00	0.43–1.00			< biTLD < monoTLD
ToM (maximum 3)	*M* (*SD*)	0.64 (0.74)	1.0 (1.00)	2.54 (0.69)	1.97 (0.89)	21.07	<0.001	(monoHFA = biHFA)
	Range	0–2	0–3	1–3	0–3			< (biTLD = monoTLD)
Verbal Working Memory	*M* (*SD*)	2.79 (1.31)	1.85 (1.72)	3.21 (0.92)	3.07 (1.05)	4.36	<0.001	All pair-wise
	Range	0–6	0–5	2–6	2–6			comparisons were n.s.
Inhibition/Selective	*M* (*SD*)	1.57 (1.60)	1.75 (2.18)	5.32 (2.28)	5.03 (1.71)	19.19	<0.001	(monoHFA = biHFA)
Attention (maximum score 10)	Range	0–4	0–7	1–10	2–8			< (biTLD = monoTLD)

We ran a series of ANOVAs with group (monoHFA, biHFA, monoTLD, biTLD) as an independent variable and morpho-syntactic abilities, ToM skills, working memory and inhibition as dependent variables. Descriptive statistics and group comparisons using Tamhane *post hoc* tests are presented in Table 2. Children with HFA scored lower than children with TLD on all measures, except for the measure of working memory. Importantly, there were no differences between monoHFA and biHFA children.

This is even more interesting, as the biTLD group scored lower compared to the monoTLD group (e.g., morpho-syntax). On ToM skills, children with HFA scored lower regardless of their language statues, yet no differences were observed between monolinguals and bilinguals.

#### Control Adult Group

In addition to the children, a control group of 18 monolingual Hebrew-speaking adults participated (12 females and 6 males). They ranged in age from 20 to 37 years (*M* = 25.9, *SD* = 4.44). Adult participants completed the pronoun task to obtain a baseline on third-person subject and object pronoun use in Hebrew.

### Materials

#### Pronoun Elicitation Task

A pronoun elicitation task tapping into the production of third-person subject and object pronouns in Hebrew was developed for this study. The task was based on previous tasks eliciting pronominal elements: the COST Action A33 tool eliciting clitics (Varlokosta et al., 2016) and the Hebrew task targeting pronouns and reflexives in the object position (Ruigendijk et al., 2010). Reflexives were not tested in the current study. The elicitation task included 12 items (6 targeting pronouns in the subject position and 6 items targeting pronouns in the object position). All targeted pronouns were singular pronouns and were carefully matched for gender in each condition: there was an equal number of items targeting feminine and masculine pronouns. The child was asked to complete a sentence based on the picture following a prompt (see (1)–(2)).

Responses for the pronouns task for children and adults were coded following the schemata presented in (1a–f) for subject pronouns and (2a–f) for object pronouns. For the purposes of our analysis, we used three categories: *Pronoun use*, which incorporates Target production (1a, 2a) and Pronoun substitution (1b, 2b); *Pronoun omission* (1c and 2c) and *Other*, which incorporated Full noun phrase, Other, No answer (1d–f; 2d–f).

(1) **PROMPT (for subject pronouns: a picture of a girl jumping in a puddle)**^3^ **:**
  ha- yalda retuva ki…
  DEF- girl wet because …
  “The girl is wet because …”

**Table:** 

	**Child’s Response**			

(1a) Target	***hi***	*kafca*	*la-shlulit*	
	she.NOM	jumped	to-the-puddle	
	“She jumped into the puddle.”			

(1b) Pronoun substitution	*daxfu*	***ota***	*la-shlulit*	
	pushed.3P.PL **she.ACC**	her	to-the-puddle	
	“Somebody pushed her in-the-puddle.”			

(1c) Pronoun omission	***e***	*kafca*	*la- shlulit*	
	**e**	jumped	to-the-puddle	
	“(She) jumped into the puddle.”			

(1d) Full noun	***yalda***	***xamuda***	*kafca*	*la-shlulit*
	**yalda.NOM**	**nice.NOM**	jumped	to-the-
				puddle
	“A nice girl jumped into the puddle.”			

(1e) Other	*mayim*	*yesh*	*po*	
	water	there	here	
	“There is water here.”			

(1f) No answer	No response
(2) **PROMPT (for object pronouns: a picture of a father lifting a baby**)^4^ **:**
  ha- tinok coxek ki …
  DEF- baby laughs because…
  “The baby is laughing because…”

**Table:** 

	**Child’s Response**			

(2a) Target	*aba*	*merim*	***oto***	
	father	lifts	him.ACC	
	The father is lifting him.			

(2b) Pronoun substitution	***hu***	*al*	*aba*	
	he.NOM	on	father	
	“He is on the father.”			

(2c) Pronoun omission	*aba*	*merim*	***e***	
	father	lifts	***e***	
	“The father is lifting.”			

(2d) Full noun	*aba*	*merim*	*et*	***ha-tinok***
	father	lifts	ACC	DEF-baby
	The father is lifting the baby.			

(2e) Other	*aba*	*po*		
	father	here		
	“The father is here.”			

(2f) No answer	No response

#### Morpho-Syntax

A shortened version of the *Hebrew LITMUS Sentence-Repetition task (SRep-30)*, based on the longer version, which comprised 56 sentences (Armon-Lotem and Meir, 2016; Meir et al., 2016), was administered as an index of children’s morpho-syntactic abilities. For more details on the morpho-syntax of the children tested in this study, see Meir and Novogrodsky (2019).

#### Theory of Mind

The total score of three ToM-based tasks was used in the current study: the “Smarties” task, a first-order false-belief task and a second-order false belief task.

In the Smarties task (the unexpected content, Perner et al., 1987), the child is shown a candy box that contains unexpected contents rather than expected sweets. Then the child is shown an unexpected object and is asked what a person who has not seen the contents of the box will say there is in the candy box. The task assesses children’s ability to consider that even if we know something, other people may have false beliefs about the same thing. For the first-order false-belief task and second-order false-belief (Wimmer and Perner, 1983; Baron-Cohen et al., 1985), a computerized film versions of the tasks (Buac and Kaushanskaya, 2019) were adapted from English to Hebrew. The first-order false-belief task assesses the ability to consider that people with beliefs, different from ours, even if false, act accordingly to those beliefs, independently from our own knowledge. The second-order false-belief task evaluates the ability to mentalize one person’s (false) belief about what another person thinks about the world. The participants were allocated a score of 1 for passing each task. Thus, ToM scores ranged from 0 to 3.

#### Working Memory

The Hebrew Backward Digit Span adapted from the Wechsler Intelligence Scale for Children (Wechsler, 1991) was used to assess children’s working memory. The children were asked to repeat digit sequences, orally, backwards. Test items consisted of two lists of digits administered for each list length, beginning with a length of two digits and increasing in length by one digit following successful repetition of at least one list of digits at a given length. The task was discontinued when the child failed at two consecutive digit sequences of the same length. The longest list length correctly repeated for each span was noted.

#### Inhibition

The Embedded Figures Task (Iluz-Cohen and Armon-Lotem, 2013) was used to test inhibition/selective attention. The task is comprised of 10 pictures (Kor, 1992). Each picture includes an embedded mouse, which the child needs to find. The ten pictures were presented in gradually increasing levels of difficulty, as a function of the amount of information in the picture, referred to as “noise” in the signal detection literature (Green and Swets, 1966). The scores on the task ranged from 0 to 10, with higher scores indicating better levels of inhibition/selective attention.

### Procedure

The study was approved by the IRB of Haifa University and the Chief Scientist of the Israeli Ministry of Education. Adult participants provided informed written consent. For children, written informed parental consent was obtained prior to participation for each child. Before each testing session, the children’s oral assent was secured. Each participant was tested in Hebrew individually, in a quiet room, in the preschool/school or at home. The bilingual children were also tested in Russian in a separate meeting. Russian and Hebrew sessions for bilingual children were counter-balanced.

## Results

### Third-Person Subject and Object Pronoun Use in Monolingual and Bilingual Children With TLD and HFA

**FIGURE 1:**
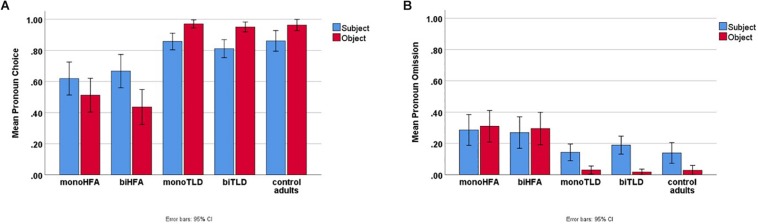
Pronoun use and pronoun omission per syntactic condition (subject vs. object) per group. **(A)** Pronoun use. **(B)** Pronoun omission.

The results for pronoun use and pronoun omission per syntactic condition per group are presented in Figure 1.

To address our first research question regarding the effects of bilingualism and HFA on the use third-person subject and object pronouns, we explored the performance of the child groups, using a generalized linear mixed logistic regression. The analyses were conducted using a statistical package SPSS 25. First, we coded children’s responses as “Pronoun Use = 1,” if a child produced a pronoun (collapsing together Target and Pronoun Substitution) and “Non-pronoun = 0,” if a response did not have a pronoun (collapsing together Pronoun Omissions and Other).

Fixed effects included Language_Status (Monolingual, Bilingual), Clinical_Status (HFA, TLD), Syntactic_Position (Subject, Object). We also included the interaction of the fixed effects into the model as predictors (Clinical_Status ^∗^ Language_Status, Clinical_Status ^∗^ Syntactic_Position, Language_Status ^∗^ Syntactic_Position, and Language_Status ^∗^ Clinical_Status ^∗^ Syntactic_Position). Participants and items were entered as random effects. The model included a random intercept for each item and a random intercept for participant.

The results indicated a strong fit of the model (C-index = 0.87). The model showed a main effect of Clinical_Status, indicating that children with HFA produced fewer pronouns overall as compared to their TLD peers. There was no significant main effect of Language_Status and no significant Language_Status ^∗^ Clinical_Status and Language_Status ^∗^ Clinical_Status ^∗^ Syntactic_Position interactions, suggesting that bilingualism does not affect the performance of children with TLD and HFA (see Table 3). Yet, there was a significant Clinical_Status ^∗^ Syntactic_Position interaction. Children with HFA produced fewer pronouns in the object position as compared to the subject position, as determined by pair-wise contrasts with an adjusted alpha-level (β = 0.22, SE = 0.08, *t* = 2.70, *p* = 0.01), for children with TLD this gap was not significant (β = 0.11, SE = 0.112, *t* = 1.06, *p* = 0.29).

**TABLE 3:** The model results for the pronoun use.

**Model term**	**Coefficient**	**SE**	***t***	***p*-value**
Intercept	0.543	1.1624	0.467	0.640
Language_Status (MONO vs. BI)	0.400	0.5807	0.689	0.491
Clinical_Status (TLD vs. ASD)	1.427	0.5037	2.834	0.005
Syntactic_Position (Subject vs. Object)	–0.497	0.3879	–1.282	0.200
Clinical_Status ^∗^ Language_Status	–0.740	0.7263	–1.019	0.309
Clinical_Status ^∗^ Syntactic_Position	2.309	0.6177	3.739	0.000
Language_Status ^∗^ Syntactic_Position	–0.870	0.5286	–1.646	0.100
Language_Status ^∗^ Syntactic_Position ^∗^ Syntactic_Position	0.677	0.8445	0.802	0.423

*Within the Language_Status, monolinguals were set as a reference line; within the Clinical_Status, the TLD group was set as a reference line; within the Syntactic_Position, the performance on the subject pronouns was taken as a reference line.*

To further investigate the patterns of performance, we analyzed “Non-pronoun” responses, which were coded as “Pronoun omission = 1” and “Other = 0” (see Figure 1B). The model showed a fair fit (C-index = 0.79). The findings for the model are presented in Table 4. The results indicated a significant main effect of Clinical_Status: children with HFA omitted pronouns more frequently than children with TLD, yet there was a significant Clinical_Status ^∗^ Language_Status interaction which stemmed from the differences between monolingual HFA and TLD children as determined by pair-wise contrasts with an adjusted alpha-level (β = 0.309, SE = 0.104, *t* = 2.957, *p* = 0.003), yet in bilingual children there were no differences between HFA and TLD with respect to pronoun omissions (β = 0.099, SE = 0.119, *t* = 0.832, *p* = 0.406).

**TABLE 4:** The model results of pronoun omission responses.

**Model term**	**Coefficient**	**SE**	***t***	***p*-value**
Intercept	0.501	0.931	0.538	0.591
Language_Status (MONO vs. BI)	–0.237	0.376	–0.631	0.528
Clinical_Status (TLD vs. TLD)	–0.664	0.331	–2.005	0.046
Syntactic_Position (Subject vs. Object)	–0.371	0.462	–0.803	0.423
Clinical_Status ^∗^ Language_Status	0.988	0.477	2.071	0.039
Clinical_Status ^∗^ Syntactic_Position	–0.266	0.475	–0.561	0.575
Language_Status ^∗^ Syntactic_Position	–0.163	0.426	–0.382	0.703
Language_Status ^∗^ Syntactic_Position ^∗^ Syntactic_Position	–0.882	0.716	–1.233	0.218

*Within the Language_Status, monolinguals were set as a reference line; within the Clinical_Status, the TLD group was set as a reference line; within the Syntactic_Position, the performance on the subject pronouns was taken as a reference line.*

To further understand the children’s data, we looked into the performance of the adults. Adult’s responses (“Pronoun use = 1” and “Non-pronoun = 0”) were entered into a binomial mixed effects logistic regression model with syntactic position as a fixed factor. Participants and items were entered as random effects, with a random intercept for each item and each participant. The results showed a significant effect of the syntactic position (β = 1.104, SE = 0.509, *t* = 2.128, *p* = 0.34) indicating that adults produced more third-person object pronouns as compared to subject pronouns (see Figure 1A).

To sum up, the results indicated a robust effect of HFA. Children with HFA produced fewer third-person pronouns as compared to their TLD peers. Moreover, the results showed that the production of third-person object pronouns was lower than subject pronouns in children with HFA. This is a striking difference compared with the adult data: more pronoun use in object positions as compared to subject positions (see Figure 1A). The results indicated that there was no significant effect of bilingualism and no significant interaction between HFA and bilingualism, showing that bilingualism does not affect pronoun use in Russian-Hebrew speaking bilinguals (see Table 3). However, bilingual children with HFA might be advantaged as compared to monolingual children with HFA by exposure to two languages and perform similarly to bilingual children with TLD, as it was the case for pronoun omissions (see Table 4).

### Prerequisites of Pronoun Use in Children With HFA and TLD

To address our second research question regarding the underlying mechanisms of third-person subject and object pronouns, we evaluated the extent to which third-person pronoun use is associated with age, non-verbal IQ, morpho-syntax, ToM, working memory and inhibition in children with HFA and TLD separately. The rational for the separate analyses for HFA and TLD groups stemmed from the results presented in the previous subsection, which indicated a robust effect of HFA, yet no effect of bilingualism on pronoun use.

We ran a step-wise regression analysis with pronoun use (subject and object, separately) as the dependent variable, and age, non-verbal IQ, morpho-syntax, ToM, working memory and inhibition as predictors. The results of the models for children with HFA are presented for third-person subject (Table 5) and for third-person object pronouns (Table 6). The analysis indicated that subject pronoun use was predicted by morpho-syntactic skills, working memory capacity and ToM skills. As for the third-person object pronoun use, only the measure of morpho-syntactic skills was found to be a predictor.

**TABLE 5:** Step-wise regression analysis for Subject Pronoun use for children with HFA (*n* = 27).

**Model**	***R***	***R*^2^**	**Coefficient**	**SE**	***t***	***p*-value**
Model 1: Morpho-syntax	0.609	0.371	4.883	1.298	3.762	0.001
Model 2: Morpho-syntax	0.730	0.533	5.529	1.165	4.747	0.000
Working Memory			–0.542	0.192	–2.830	0.009
Model 3: Morpho-syntax	0.803	0.644	4.033	1.187	3.399	0.003
Working Memory			–0.595	0.172	–3.452	0.002
ToM			0.892	0.341	2.618	0.016

**TABLE 6:** Step-wise regression analysis for Object Pronoun use for children with HFA (*n* = 27).

**Model**	***R***	***R*^2^**	**Coefficient**	**SE**	***t***	***p*-value**
Model 1: Morpho-syntax	0.625	0.391	4.753	1.211	3.924	0.001

Turning to the TLD groups for a comparison, the results of a step-wise regression analysis with pronoun use, separately conducted for Subject and Object pronouns, with age, non-verbal IQ, morpho-syntax, ToM, working memory and inhibition as predictors (see Tables 7, 8), showed that only age was a significant predictor for both types of third-person pronouns.

**TABLE 7:** Step-wise regression analysis for Subject Pronoun use for children with TLD (*n* = 58).

**Model**	***R***	***R*^2^**	**Coefficient**	**SE**	***t***	***p*-value**
Model 1: Age	0.454	0.206	−0.050	0.013	−3.811	0.000

**TABLE 8:** Step-wise regression analysis for Object Pronoun use for children with TLD (*n* = 58).

**Model**	***R***	***R*^2^**	**Coefficient**	**SE**	***t***	***p*-value**
Model 1: Age	0.343	0.118	0.015	0.005	2.737	0.008

To summarize, while for TLD children only age played a role in explaining their performance, for children with HFA different linguistic and cognitive abilities affected their pronoun use

## Discussion

The current study was two-fold. First, it assessed effects of bilingualisms and HFA on third-person subject and object production in Hebrew, using a structured elicitation task. Second, it evaluated the underlying prerequisites of third-person subject and object pronoun use in monolingual and bilingual children with HFA and TLD. We considered the role of linguistic and non-linguistic factors.

Previous studies have reported mixed findings with respect to pronoun use in children with HFA. Similarly, previous findings bring inconclusive on pronoun use in bilingual children. Thus, the comparison of four child groups (monoHFA, biHFA, monoTLD and biTLD) was intended to provide insights into the interaction of these two precursors of pronoun use in typical and atypical populations.

### Separate and Combined Effects of Bilingualism and HFA on Third-Person Pronoun Subject and Object Pronoun Use

The results of the current study indicated a robust effect of HFA, no effect of bilingualism and no interaction between bilingualism and HFA.

Before discussing the effects of HFA and bilingualism, we will briefly remind the mechanisms of third-person subject and object pronouns in Hebrew. Hebrew is the language spoken by all children tested in the study, in the case of bilinguals it is the Societal Language. Bilingual children in the current study all spoke Russian, as their Heritage Language. As outlined in Subsection 1.3, despite being typologically different languages, Russian and Hebrew show a number of similarities in their pronominal systems and licensing of overt vs. null pronouns. This should be kept in mind when discussing the effects of bilingualism for the current sample. Hebrew is a “partial pro-drop language,” which licenses subject pro-drop of first- and second-object pronouns morpho-syntactically, yet when it comes to third-person pronouns, which are studied in the current study, their omissions are governed by discourse-drop mechanisms. Interestingly, although pronouns can be omitted in both positions based on discourse conditions, the results of the adults in the current study showed a high rate of overt pronouns in the targeted subject and object positions. Furthermore, adults were found to produce third-person object pronouns at a higher rate than third-person subject pronouns (compare 96% vs. 86%, respectively). The data for third-person object pronouns are in line with previous studies showing very low omissions of third-person object pronouns both in adults and in children (Varlokosta et al., 2016).

First, we will discuss the effect of bilingualism on third-person pronouns use. No effect of bilingualism was predicted to be found, based on similarities of the pronominal systems of Hebrew and Russian and similarities in the licensing mechanisms of null pronominal elements in the two languages. The prediction has been borne out. Furthermore, this was confirmed for bilingual children with TLD and for bilingual children with HFA, as evident in the lack of interaction between HFA and bilingualism. Bilingual children seem to pair up with their monolingual controls. Our study does not confirm previous studies, which showed differences between monolinguals and bilinguals on pronoun use (Hulk and Müller, 2000; Paradis and Navarro, 2003; Sorace, 2005; Serratrice, 2007; Sorace and Serratrice, 2009). Yet, it should be noted that in many of the previous studies, pronominal systems in the two languages varied: (1) with respect to lexical realization of pronominal elements (clitic pronouns vs. strong/weak pronouns) and (2) with respect to the licensing of pronominal elements (e.g., subject [+pro-drop] like Italian and subject [-dro-drop] like English). Thus, our study demonstrates that the acquisition of a pronominal system in the Societal Language might be facilitated when the two languages of a bilingual child show similarities in their pronominal systems.

Turning to the effect of HFA, our findings showed its robust effect on third-person pronoun use. Previous research showed mixed evidence of the use of pronominal elements in children with HFA. In the current study, children with HFA (regardless of their language status: monolingual or bilingual) produced fewer third-person pronouns in the subject position and in the object position as compared with their TLD peers. Interestingly, children with HFA produced fewer target third-person object pronouns as compared to subject pronouns. These findings are in a striking contrast as compared to the adult data, which presented the reverse pattern. Monolingual Hebrew-speaking adults produced more target third-person pronoun in the object position as compared to the subject position. In children with TLD, no statistical differences were found between subject and object pronouns. However, when looking at the descriptive data (of the children with TLD), the patterns are similar to those of adult data (for monoTLD children: 97% vs. 86%; for biTLD: 96% vs. 86%, respectively).

Furthermore, looking into omissions of subject and object pronouns, the results showed that children with HFA omitted pronouns more frequently than children with TLD. There was a significant interaction between HFA and bilingualism. This indicates that there was a significant difference in omissions of pronouns in monolingual children with and without HFA, while there was no significant difference in bilingual children with and without HFA. This gap can be explained as a bilingual advantage for children with HFA, suggesting that experiencing two languages with similar linguistic features support their acquisition (also see Roeper, 2012).

Although, third-person subject and object pronouns can be omitted in Hebrew based on discourse properties, the adults’ data showed an asymmetry between third-person subject and object use of null pronominal elements. Adults omitted more third-person subject pronouns than object pronouns. However, children with HFA showed an opposite pattern with omitting more third-person object pronouns as compared to third-person subject pronouns. We will return to this point when discussing the prerequisites of third-person subject and object pronoun use in the next subsection.

### Prerequisites of Third-Person Subject and Object Pronoun Use Among Children With HFA and TLD

Pronouns use is a complex linguistic phenomenon, which requires linguistic and non-linguistic skills. Previous studies attributed problems with third-person pronouns to difficulties with morpho-syntax, especially in languages in which pronominal elements are mapped onto clitic pronouns (e.g., French speaking children with HFA: Durrleman and Delage, 2016; Tuller et al., 2017; Prévost et al., 2018; Greek speaking children with HFA: Terzi et al., 2012). In contrast, some studies claimed that poor use of pronouns is related to deficit in ToM (Kuijper et al., 2015), poor working memory (Koster et al., 2011; Marinis and Chondrogianni, 2011) and inhibition (Kuijper et al., 2015).

In the current study, we evaluated linguistic and non-linguistic factors associated with third-person pronoun use. As a group, children with monoHFA and biHFA scored lower than their monolingual and bilingual peers with TLD. This was observed for morpho-syntax, ToM and inhibition, while no differences were found between children with HFA and TLD on working memory. Importantly, no significant difference was shown between monoHFA and biHFA across these measures. Thus, the potential prerequisites for pronoun use are beyond language status (monolingual or bilingual).

Based on the hypothesis that linguistic and non-linguistic components might be associated with pronoun use, we explored the role of morpho-syntax, ToM, working memory, and inhibition on pronoun use among children with HFA and TLD. This model follows the concept of a direct comparison of different domains within the same group of children with ASD. For example, Kuijper et al. (2015) showed that the use of referential expressions in children with ASD was related to ToM and to a lesser degree with inhibition and working memory capacity in narrative production tasks. However, the study did not assess linguistic skills of the children.

The results of the current study showed that different mechanisms might be associated with third-person subject and object pronouns of children with HFA. Third-person subject pronoun use was predicted by morpho-syntactic skills, working memory capacity and ToM skills. As for the third-person object pronoun use, only the index of morpho-syntactic skills was found to be a predictor. Morpho-syntactic abilities as measured by a sentence repetition task was the strongest predictor of pronoun use of the HFA group. It is important to note that children with HFA did not have problems with gender (*he, she*), number (*he, them*), person (*he, you*) or case (*he, him*) features. This pattern is in agreement with previous studies. For example, Novogrodsky (2013) reported only 2% morpho-syntactic errors (gender, number, person and agreement) for third-person subject pronouns (e.g., *he* for *they; she* for *he*) of monolingual English speaking children with HFA. The strong relationship between morpho-syntax and pronoun use is explained in previous research, by classifying subgroups of children with ASD. Specifically, it has been shown that some children with ASD with comorbid Language Disorder have problems with clitic pronouns, while children with ASD with intact morpho-syntax do not (Durrleman and Delage, 2016; Tuller et al., 2017; Prévost et al., 2018). Similarly, problems with clitic pronouns in children with ASD speaking Greek were attributed to syntax (Terzi et al., 2012), indicating the syntactic nature of problems.

We suggest that a similar pattern is observed in the current study. The children who scored low on the Sentence Repetition task had Language Disorder (for more details see Meir and Novogrodsky, 2019) and are the ones who scored low on the pronoun use task. This can also explain the asymmetry between production of subject and object third-person pronouns in the group of children with HFA. This asymmetry was in sharp contrast with the adult data in which object pronoun omissions are not attested. The low rates of object pronoun production in children with HFA as compared to subject pronouns might be attributed to problems with morpho-syntax *per se* rather than sensitivity to discourse distributions of null and overt pronouns. It is plausible to suggest that children with HFA who exhibit impaired morpho-syntax have incomplete argument structure representations, as previously suggested for children with Developmental Language Disorder (Thordardottir and Weismer, 2002). Furthermore, a recent study on pronoun use in children with HFA with intact morpho-syntax showed no problems with pronoun use (Terzi et al., 2019). Thus, the relationship between object pronoun omission and morpho-syntactic impairment awaits future studies. Comparing pronoun production of children with HFA with and without comorbid Language Disorder will shed light on this notion.

Now let us turn to the involvement of ToM and working memory in the production of third-person subject pronouns. Here we would like to expand our discussion on the low predictive power of ToM in third-person subject pronoun use, and the lack of predictive power for third-person object pronouns. One explanation lies in the nature of the pronoun elicitation task. The task used in the current study evaluates pronoun use at a sentence level, which might explain the strong relations between pronoun production and scores on the sentence repetition task. This is similar to previous studies that used elicitation tasks in order to explore syntactic structures (Terzi et al., 2012). However, the characteristics of the current task require also discourse knowledge, i.e., evaluating whether or not a referent is familiar to the listener. The integration of these components requires the child to represent the interlocutor’s knowledge, which is part of ToM capacity and is agreed to be impaired in children with HFA (Baron-Cohen, 1988; Tager-Flusberg and Anderson, 1991). Thus, ToM is potentially required, although it was found not to have a large effect. Another explanation for the low explanatory power of ToM in the current study is the prerequisites of the development of ToM skills. Numerous studies demonstrated a link between ToM development and morpho-syntax (for an overview see De Villiers, 2007). Our inclination is to suggest that ToM is involved in pronoun use; yet, morpho-syntactic development plays an important role in both ToM and in pronoun use.

Interestingly, working memory, as measured by Backward Digit Span also predicted performance on third-person subject pronouns. This has been previously attested for children with HFA and TLD (Kuijper et al., 2015). Studies linking third-person pronoun use to the capacity of working memory were mainly reported for pronoun use in narratives. Our study showed that a small portion of the variance is explained by working memory even at a local sentence level. Thus, sufficient verbal working memory capacity is one the prerequisites of pronoun use. A speaker needs to maintain the discourse referent activated and accessible, which implicates verbal working memory. Additionally, it is plausible to suggest that verbal working memory capacity is related to language skills, which were found to explain the lion’s portion of the variance in the production of third-person subject and object pronouns in the current study. Yet, the latter explanation will not hold for this sample of children as verbal working memory capacity was found not to be linked to morpho-syntactic skills (for more details see Meir and Novogrodsky, 2019). Furthermore, working memory explained the variance of third-person subject pronoun use, which is argued to be licensed by discourse-pragmatics (Ariel, 2001), and it did not explain the variance of object pronoun omissions. Thus, it is plausible to suggest that working memory is related to discourse-pragmatic properties of pronoun use. The assumption remains open for future studies.

Finally, our study did not confirm a link between pronoun use and inhibition, although it was suggested that inhibition is a potential factor affecting pronoun use. The idea is that the child must block the form that is optimal from his/her own perspective in order to produce the form that is optimal for the listener (Kuijper et al., 2015). The involvement of inhibition in ToM skills and pronoun use needs further examination using different tasks tapping into inhibition skills, possibly tasks involving verbal inhibition rather than visual inhibition as it has been used in the current study.

## Conclusion

Our study demonstrated that bilingual children with and without HFA show similar performance to their monolingual peers on third-person subject and object pronoun use. The results indicated that bilinguals perform similarly to monolinguals when the pronominal system in the two language of bilinguals show similarities, like in the case of Russian-Hebrew bilinguals. The findings have important implication for understanding the interface of bilingualism and HFA because it demonstrates that bilingualism is not harmful for children with HFA.

Children with HFA, regardless of their language status, showed low scores of third-person subject and object pronouns use. Interestingly, they produced more third-person subject pronouns compared with object pronouns, which is in sharp contrast with the adults’ data, which showed the opposite picture: no omissions of pronouns in the object position, while higher rates of null pronominal elements in the subject position.

The current study demonstrates that third-person subject and object pronoun use is a complex task, which requires integration of multiple linguistic and non-linguistic components. In children with HFA (regardless of their language status), third-person subject and object pronoun use is largely dependent on morpho-syntactic abilities; and additionally, third-person subject pronoun use is linked to ToM skills and working memory.

## Ethics Statement

The study was approved by the IRB of the University of Haifa and the Israeli Ministry of Education.

## Author Contributions

NM and RN discussed the conceptual idea, designed the pronoun task, and wrote the manuscript. NM analyzed the data.

## Conflict of Interest

The authors declare that the research was conducted in the absence of any commercial or financial relationships that could be construed as a potential conflict of interest.

## Footnotes

1 The three types of pronominal elements vary in their syntactic distribution, morphology, semantics and prosody (for more detail on the distinction between strong pronouns, weak pronouns and pronominal clitics, see Cardinaletti and Starke, 1996). In many languages, weak and strong pronouns are homophonous. Clitic pronouns are bound morphemes (e.g., in French *Je* ***l****’aime* “I love him”), while weak and strong pronouns are both free morphemes (e.g., in English: *I love* ***you***). Strong pronouns can be coordinated (e.g., in English: *I love* ***him*** *and* ***her***), while weak pronouns and pronominal clitics cannot. Clitics cannot bear lexical word stress, whereas strong and weak pronouns can.

2 Languages fall into three categories with respect to allowing null pronominal elements, (1) pro-drop type languages in which subject and object null elements are conditioned by agreement, i.e., respectively by subject-verb agreement (e.g., Italian, Spanish) or object-verb-agreement (e.g., Pashto); (2) topic-drop type languages in which subject and object drops are conditioned by an empty sentence-initial position (e.g., German); and (3) discourse-drop type languages in which null pronominal elements are not conditioned by syntactic restrictions (for more details see Sigurðsson, 2011).

3 The pictures were taken from educational websites and adapted for the target stimuli: https://www.mycutegraphics.com/graphics/school/girl-jumping-clip-art.html

4 The pictures were taken from educational websites: https://classroomclipart.com/clipart-view/Clipart/Baby/dad-lifting-a-baby-with-smile-clipart-5122_jpg.htm

## References

[B1] Abutbul-Oz H. Armon-Lotem S. Walters J. (2012). *Bilingual Parents Questionnaire (BIPAQ).* Ramat Gan: Bar-Ilan University.

[B2] American Psychiatric Association (2013). *Diagnostic and Statistical Manual of Mental Disorders*, 5th Edn Washington, D.C: American Psychiatric Association.

[B3] Ariel M. (2001). “Accessibility theory: an overview,” In *Text representation: Linguistic and psycholinguistic aspects*, eds Sanders T. Schilperoord J. Spooren W. (Amsterdam: John Benjamins Publishing Company), 29–87.

[B4] Ariel M. (2004). Accessibility marking: discourse functions, discourse profiles, and processing cues. *Discourse processes* 37 91–116. 10.4324/9781315046105-2

[B5] Armon-Lotem S. (2008). “Subject use and the acquisition of verbal agreement in Hebrew,” in *The Acquisition of Verbs and Their Grammar: The Effect of Particular Languages*, eds Gagarina N. Gülzow I. (Dordrecht: Springer), 45–67. 10.1007/1-4020-4335-x_2

[B6] Armon-Lotem S. Meir N. (2016). Diagnostic accuracy of repetition tasks for the identification of specific language impairment (SLI) in bilingual children: evidence from Russian and Hebrew. *Int. J. Lang. Commun. Disord.* 51 715–731. 10.1111/1460-6984.12242 26990037

[B7] Arnold J. E. Bennetto L. Diehl J. J. (2009). Reference production in young speakers with and without autism: effects of discourse status and processing constraints. *Cognition* 110 131–146. 10.1016/j.cognition.2008.10.016 19111285 PMC3668432

[B8] Barbosa P. P. (2011). Pro-drop and theories of pro in the minimalist program part 1: consistent null subject languages and the pronominal-agr hypothesis. *Lang. Linguist. Compass* 5 551–570. 10.1111/j.1749-818x.2011.00293.x

[B9] Baron-Cohen S. (1988). Social and pragmatic deficits in autism: cognitive or affective? *J. Autism Dev. Disord.* 18 379–402. 10.1007/bf02212194 3049519

[B10] Baron-Cohen S. (2000). “Theory of mind and autism: a fifteen-year review,” in *Understanding other Minds: Perspectives from Developmental Cognitive Neuroscience*, eds Baron–Cohen S. Tager–Flusberg H. Cohen D. J. (Oxford: Oxford University Press), 3–20.

[B11] Baron-Cohen S. Leslie A. M. Frith U. (1985). Does the autistic child have a “theory of mind”? *Cognition* 21 37–46. 10.1016/0010-0277(85)90022-8 2934210

[B12] Berman R. Lustigman L. (2012). “HARSP: a developmental language profile for Hebrew,” in *Communication Disorders Across Languages*, eds Ball M. J. Crystal D. Fletcher P. (Bristol: Multilingual Matters), 43–76. 10.21832/9781847696397-006

[B13] Berman R. A. (1985). “The acquisition of Hebrew,” In, The crosslinguistic study of language acquisition, ed Slobin D. I. (Hillsdale, NJ, US: Lawrence Erlbaum Associates, Inc.), 255–371.

[B14] Bishop D. V. (2003). “Autism and specific language impairment: categorical distinction or continuum,” in *Autism: Neural Basis and Treatment Possibilities*, eds Bock G. R. Goode J. A. (Chichester: Novartis Foundation), 213–234. 10.1002/0470869380.ch13 14521195

[B15] Buac M. Kaushanskaya M. (2019). Predictors of Theory of Mind performance in bilingual and monolingual children. *Int. J. Billing.* 10.1177/1367006919826866 PMC5041596 27179914

[B16] Cardinaletti A. Starke M. (1996). “Deficient pronouns: a view from Germanic. Studies in comparative Germanic syntax,” in *Studies in Comparative Germanic Syntax*, eds Thräinsson H. Epstein S. D. Peter S. (Dordrecht: Kluwer Academic Publishers), 221–265.

[B17] Chondrogianni V. (2015). “Production and comprehension of pronouns and reflexives in atypical populations,” in *The Acquisition of Reference*, eds Serratrice L. Allen S. E. M. (Amsterdam: John Benjamins Pub Co.), 285–309. 10.1075/tilar.15.12cho

[B18] De Villiers J. (2007). The interface of language and theory of mind. *Lingua* 117 1858–1878. 10.1016/j.lingua.2006.11.006 17973025 PMC2000852

[B19] Doron E. (1999). “V-movement and VP ellipsis,” in *Fragments: Studies in ellipsis and gapping*, eds Lappin S. Benmamoun E. (Oxford: Oxford University Press), 124–1400.

[B20] Durrleman S. Delage H. (2016). Autism spectrum disorder and specific language impairment: overlaps in syntactic profiles. *Lang. Acquisit.* 23 361–386. 10.1080/10489223.2016.1179741

[B21] Eigsti I. M. de Marchena A. B. Schuh J. M. Kelley E. (2011). Language acquisition in autism spectrum disorders: a developmental review. *Res. Autism Spectr. Disord.* 5 681–691. 10.1016/j.rasd.2010.09.001

[B22] Fichman S. Altman C. (2019). Referential cohesion in the narratives of bilingual and monolingual children with typically developing language and with specific language impairment. *J. Speech Lang. Hear. Res.* 62 123–142. 10.1044/2018_JSLHR-L-18-0054 30950755

[B23] Franks S. (1995). *Parameters of Slavic Morphosyntax*. Oxford: Oxford University Press.

[B24] Gordishevsky G. Avrutin S. (2003). Subject and object omissions in child Russian. *Proc. Israel Assoc. Theor. Linguist.* 19 1–26.

[B25] Green D. A. Swets J. A. (1966). *Signal Detection Theory and Psychophysics.* New York, NY: Wiley.

[B26] Grimshaw J. Samek-Lodovici V. (1998). “Optimal subjects and subject universals,” in *Is the Best Good Enough? Optimality and Competition in Syntax*, eds Barbosa P. Fox D. Hagstrom P. McGinnis M. Psetsky D. (Cambridge: MIT Press), 193–219.

[B27] Gundel J. K. Ntelitheos D. Kowalsky M. (2006). Children’s use of referring expressions: some implications for theory of mind. *Pap. Linguist.* 48 1–22.

[B28] Hacohen A. Schaeffer J. (2007). Subject realization in early Hebrew/English bilingual acquisition: the role of crosslinguistic influence. *Biling. Lang. Cogn.* 10 333–344. 10.1017/s1366728907003100

[B29] Hill E. L. (2004). Executive dysfunction in autism. *Trends Cogn. Sci.* 8 26–32. 10.1016/j.tics.2003.11.003 14697400

[B30] Holmberg A. Nayudu A. Sheehan M. (2009). Three partial null-subject languages: a comparison of brazilian portuguese. Finnish and marathi. *Stud. Linguist.* 63 59–97. 10.1111/j.1467-9582.2008.01154.x

[B31] Hulk A. Müller N. (2000). Bilingual first language acquisition at the interface between syntax and pragmatics. *Biling. lang. Cogn.* 3 227–244. 10.1017/s1366728900000353

[B32] Iluz-Cohen P. Armon-Lotem S. (2013). Language proficiency and executive control in bilingual children. *Biling. Lang. Cogn.* 16 884–899. 10.1017/s1366728912000788

[B33] Ivanova-Sullivan T. (2014). *Theoretical and Experimental Aspects of Syntax-Discourse Interface in Heritage Grammars.* Boston: Brill Publishers.

[B34] Kjelgaard M. M. Tager-Flusberg H. (2001). An investigation of language impairment in autism: Implications for genetic subgroups. *Lang. Cogn. Process.* 16 287–308. 10.1080/01690960042000058 16703115 PMC1460015

[B35] Kor P. (1992). *Where is the Mouse? Zmora Bitan.* Tel Aviv: Hebrew.

[B36] Koster C. Hoeks J. Hendriks P. (2011). “Comprehension and production of subject pronouns: Evidence for the asymmetry of grammar,” in *Production-Comprehension Asymmetries in Child Language*, eds Grimm A. Müller A. Hamann C. Ruigendijk E. (Berlin: De Gruyter), 99–122.

[B37] Kuijper S. J. Hartman C. A. Hendriks P. (2015). Who is he? Children with ASD and ADHD take the listener into account in their production of ambiguous pronouns. *PloS One* 10:e0132408. 10.1371/journal.pone.0132408 26147200 PMC4492581

[B38] Laenzlinger C. Shlonsky U. (1997). Weak pronouns as LF clitics: clustering and adjacency effects in the pronominal systems of German and Hebrew. *Stud. Linguist.* 51 154–185. 10.1111/1467-9582.00020

[B39] Landau I. (2018). Missing objects in Hebrew: argument ellipsis, not VP ellipsis. *Glossa* 3 1–37.

[B40] Lord C. Rutter M. DiLavore P. S. Risi S. (2003). *Autism Diagnostic Observation Schedule: ADOS.* Los Angeles: Western Psychological Services.

[B41] Marinis T. Chondrogianni V. (2011). Comprehension of reflexives and pronouns in sequential bilingual children: do they pattern similarly to L1 children, L2 adults, or children with specific language impairment? *J. Neurolinguistics* 24 202–212. 10.1016/j.jneuroling.2010.02.009

[B42] Martinussen R. Hayden J. Hogg-Johnson S. Tannock R. (2005). A meta-analysis of working memory impairments in children with attention-deficit/hyperactivity disorder. *J. Am. Acad. Child Adolesc. Psychiatry* 44 377–384. 15782085 10.1097/01.chi.0000153228.72591.73

[B43] Meir N. Walters J. Armon-Lotem S. (2016). Disentangling bilingualism from SLI using sentence repetition tasks: the impact of L1 and L2 properties. *Int. J. Biling.* 20 421–452. 10.1177/1367006915609240

[B44] Meir N. Novogrodsky R. (2019). Syntactic abilities and verbal memory in monolingual and bilingual children with High Functioning Autism (HFA). *First Lang.* 10.1177/0142723719849981

[B45] Melnik N. (2007). “Extending partial pro-drop in Modern Hebrew: a comprehensive analysis,” in *Proceedings of the 14th International Conference on Head-Driven Phrase Structure Grammar*, ed. Müller S. (Redwood City: Stanford Publications), 173–193.

[B46] Novogrodsky R. (2013). Subject pronoun use by children with autism spectrum disorders (ASD). *Clin. Linguist. Phon.* 27 85–93. 10.3109/02699206.2012.742567 23294224

[B47] Novogrodsky R. Edelson L. R. (2016). Ambiguous pronoun use in narratives of children with Autism Spectrum Disorders. *Child Lang. Teach. Ther.* 32 241–252. 10.3109/02699206.2012.742567 23294224

[B48] Paradis J. Crago M. Genesee F. (2006). Domain-general versus domain-specific accounts of specific language impairment: evidence from bilingual children’s acquisition of object pronouns. *Lang. Acquisit.* 13 33–62. 10.1207/s15327817la1301_3

[B49] Paradis J. Navarro S. (2003). Subject realization and crosslinguistic interference in the bilingual acquisition of Spanish and English: what is the role of the input? *J. Child Lang.* 30 371–393. 10.1017/s0305000903005609 12846302

[B50] Perner J. Leekam S. R. Wimmer H. (1987). Three-year-olds’ difficulty with false belief: the case for a conceptual deficit. *Br. J. Dev. Psychol.* 5 125–137. 10.1111/j.2044-835x.1987.tb01048.x 10536224

[B51] Prévost P. (2015). “Elicited Production of Object Clitics,” in *Methods for Assessing Multilingual Children: Disentangling Bilingualism from Specific Language Impairment*, eds Armon-Lotem S. de Jong J. Meir N. (Bristol: Multilingual Matters), 55–75. 10.21832/9781783093137-005

[B52] Prévost P. Tuller L. Zebib R. Barthez M. A. Malvy J. Bonnet-Brilhault F. (2018). Pragmatic versus structural difficulties in the production of pronominal clitics in French-speaking children with autism spectrum disorder. *Autism Dev. Lang. Impair.* 3:2396941518799643.

[B53] Raven J. (1998). *Ravens Coloured Progressive Matrices.* San Antonio, TX: Psychological Corporation.

[B54] Roeper T. (2012). Minimalism and bilingualism: how and why bilingualism could benefit children with DLD. *Biling. Lang. Cogn.* 15 88–101. 10.1017/s1366728911000605

[B55] Rom A. Dgani R. (1985). Acquiring case-marked pronouns in Hebrew: the interaction of linguistic factors. *J. Child lang.* 12 61–77. 10.1017/s0305000900006231 3980608

[B56] Ruigendijk E. Friedmann N. Novogrodsky R. Balaban N. (2010). Symmetry in comprehension and production of pronouns: a comparison of German and Hebrew. *Lingua* 120 1991–2005. 10.1016/j.lingua.2010.02.009

[B57] Schaeffer I. Shalom D. B. (2008). “On child subjects in a partially pro-drop language,” in *Current Issues in Generative Hebrew Linguistics*, eds Armon-Lotem S. Danon G. Rothstein S. D. (Amsterdam: John Benjamins Publishing House, 245–266.

[B58] Schroeder S. R. (2018). Do bilinguals have an advantage in theory of mind? A meta-analysis. *Front. Commun.* 3:36 10.3389/fcomm.2018.00036

[B59] Serratrice L. (2007). Referential cohesion in the narratives of bilingual English-Italian children and monolingual peers. *J. Pragmat.* 39 1058–1087. 10.1016/j.pragma.2006.10.001

[B60] Serratrice L. Sorace A. Paoli S. (2004). Crosslinguistic influence at the syntax–pragmatics interface: subjects and objects in English–Italian bilingual and monolingual acquisition. *Biling. Lang. Cogn.* 7 183–205. 10.1017/s1366728904001610

[B61] Shlonsky U. (2009). Hebrew as a partial null-subject language. *Stud. Linguist.* 63 133–157. 10.1111/j.1467-9582.2008.01156.x

[B62] Sigurðsson H. Á. (2011). Conditions on argument drop. *Linguist. Inq.* 42 267–304. 10.1162/ling_a_00042

[B63] Sorace A. (2005). “Selective optionality in language development,” in *Syntax and Variation: Reconciling the Biological and the Social*, eds Cornips L. Corrigan K. (Amsterdam: John Benjamins), 46–111.

[B64] Sorace A. (2016). Referring expressions and executive functions in bilingualism. *Linguist. Approaches Biling.* 6 669–684. 10.1075/lab.15055.sor

[B65] Sorace A. Serratrice L. (2009). Internal and external interfaces in bilingual language development: beyond structural overlap. *Int. J. Biling.* 13 195–210. 10.1177/1367006909339810

[B66] Sorace A. Serratrice L. Filiaci F. Baldo M. (2009). Discourse conditions on subject pronoun realization: testing the linguistic intuitions of older bilingual children. *Lingua* 119 460–477. 10.1016/j.lingua.2008.09.008

[B67] Sukenik N. Friedmann N. (2018). ASD Is Not DLI: individuals with autism and individuals with syntactic DLI show similar performance level in syntactic tasks, but different error patterns. *Front. Psychol.* 9:279. 10.3389/fpsyg.2018.00279 29670550 PMC5894483

[B68] Tager-Flusberg H. (1995). Once upon a ribbit’: stories narrated by autistic children. *Br. J. Dev. Psychol.* 13 45–59. 10.1111/j.2044-835x.1995.tb00663.x

[B69] Tager-Flusberg H. (2016). Risk factors associated with language in autism spectrum disorder: clues to underlying mechanisms. *J. Speech Lang. Hear. Res.* 59 143–154. 10.1044/2015_JSLHR-L-15-0146 26502110 PMC4867927

[B70] Tager-Flusberg H. Anderson M. (1991). The development of contingent discourse ability in autistic children. *J. Child. Psychol. Psychiatry* 32 1123–1134. 10.1111/j.1469-7610.1991.tb00353.x 1838537

[B71] Terzi A. Marinis T. Francis K. Kotsopoulou A. (2012). “Crosslinguistic differences in autistic children’s comprehension of pronouns: English vs. Greek,” in *Proceedings of the 36th Annual Boston University Conference on Language Development*, eds Biller A. K. Chung E. Y. Kimball A. E. (Somerville, MA: Cascadilla Press), 607–619.

[B72] Terzi A. Marinis T. Zafeiri A. Francis K. (2019). Subject and object pronouns in high-functioning children with ASD of a null subject language. *Front. Psychol.* 10:1301. 10.3389/fpsyg.2019.01301 31231286 PMC6568273

[B73] Testelets Y. (2003). “Are there Strong and Weak Pronouns in Russian?,” in *Formal Approaches to Slavic Linguistics The Amherst Meeting 2002*, Vol. 11 eds Browne W. Kim J. Y. Partee B. H. Rothstein R. A. (Washington, WA.: Michigan Slavic Publications), 515–538.

[B74] Thordardottir E. T. Weismer S. E. (2002). Verb argument structure weakness in specific language impairment in relation to age and utterance length. *Clin. Linguist. Phon.* 16 233–250. 10.1080/02699200110116462 12148158

[B75] Tsimpli I. M. Peristeri E. Andreou M. (2016). Narrative production in monolingual and bilingual children with specific language impairment. *Appl. Psycholinguist.* 37 195–216. 10.1017/s0142716415000478

[B76] Tuller L. Ferré S. Prévost P. Barthez M. A. Malvy J. Bonnet-Brilhault F. (2017). “The effect of computational complexity on the acquisition of French by children with ASD,” in *Innovative Investigations of Language in Autism Spectrum Disorder*, ed. Naigle L. (Berlin: de Gruyter), 115–140. 10.1037/15964-007

[B77] Varlokosta S. Belletti A. Costa J. Friedmann N. Gavarró A. Grohmann K. K. (2016). A cross-linguistic study of the acquisition of clitic and pronoun production. *Lang. Acquisit.* 23 1–26. 10.1080/10489223.2015.1028628

[B78] Wechsler D. (1991). *WISC-III: Wechsler Intelligence Scale for Children: Manual.* San Antonio, TX: Psychological Corporation.

[B79] Welterlin A. LaRue R. H. (2007). Serving the needs of immigrant families of children with autism. *Disabil. Soc.* 22 747–760. 10.1080/09687590701659600

[B80] Wimmer H. Perner J. (1983). Beliefs about beliefs: representation and constraining function of wrong beliefs in young children’s understanding of deception. *Cognition* 13 103–128. 10.1016/0010-0277(83)90004-5 6681741

[B81] Yirmiya N. Erel O. Shaked M. Solomonica-Levi D. (1998). Meta-analyses comparing theory of mind abilities of individuals with autism, individuals with mental retardation, and normally developing individuals. *Psychol. Bull.* 124 283–307. 10.1037//0033-2909.124.3.283 9849110

[B82] Yu B. (2013). Issues in bilingualism and heritage language maintenance: Perspectives of minority-language mothers of children with autism spectrum disorders. *Am. J. Speech Lang. Pathol.* 22 10–24. 10.1044/1058-0360(2012/10-0078) 23071196

